# Predefined-Time Stability/Synchronization of Coupled Memristive Neural Networks With Multi-Links and Application in Secure Communication

**DOI:** 10.3389/fnbot.2021.783809

**Published:** 2021-12-24

**Authors:** Hui Zhao, Aidi Liu, Qingjié Wang, Mingwen Zheng, Chuan Chen, Sijie Niu, Lixiang Li

**Affiliations:** ^1^Shandong Provincial Key Laboratory of Network Based Intelligent Computing, School of Information Science and Engineering, University of Jinan, Jinan, China; ^2^School of Mathematics and Statistics, Shandong University of Technology, Zibo, China; ^3^School of Cyber Security, Qilu University of Technology (Shandong Academy of Sciences), Jinan, China; ^4^State Key Laboratory of Networking and Switching Technology, Beijing University of Posts and Telecommunications, Beijing, China

**Keywords:** predefined-time synchronization, coupled memristive neural networks, multi-links topology, secure communication scheme, Lyapunov function

## Abstract

This paper explores the realization of a predefined-time synchronization problem for coupled memristive neural networks with multi-links (MCMNN) via nonlinear control. Several effective conditions are obtained to achieve the predefined-time synchronization of MCMNN based on the controller and Lyapunov function. Moreover, the settling time can be tunable based on a parameter designed by the controller, which is more flexible than fixed-time synchronization. Then based on the predefined-time stability criterion and the tunable settling time, we propose a secure communication scheme. This scheme can determine security of communication in the aspect of encrypting the plaintext signal with the participation of multi-links topology and coupled form. Meanwhile, the plaintext signals can be recovered well according to the given new predefined-time stability theorem. Finally, numerical simulations are given to verify the effectiveness of the obtained theoretical results and the feasibility of the secure communication scheme.

## 1. Introduction

With the development of information technology and the higher requirement of information transmission, it is vital to ensure the security of communication. The field of secure communication has also attracted a large number of scientific researchers. Meanwhile, some important scientific research achievements have been obtained in the aspect of secure communication based on the synchronization performance of a chaotic system (Tao and Chua, 1997; Feki, 2003; Femat et al., 2004; Zheng et al., 2009). The types of chaotic systems include simple three-dimension chaotic systems, complex dynamical networks, and general neural networks, etc. Similar to a general neural network, the memristive neural network is also a kind of chaotic system, but differs in that the parameters of MNN are state-dependent. Memristor was first proposed by Chua (1971). Unlike ordinary resistors, which have fixed resistance values, the memristor is nonlinear and its value is not unique; the memristor is also considered to be the electronic equivalent of the synapse. As the memristor is often used to mimic the synapse, the model of MNN is widely applied in associative memory, next-generation computers, and powerful brain-like “neural” computers. MNN is the more realistic model for the description of real neural systems. Moreover, MNN with a coupling term is more suitable for real complex neural networks, which is known as a coupled memristive neural network (CMNN). Therefore, there has been an upsurge in the study of dynamic behavior based on the model of CMNN. In addition, the main considered problems also include secure transmission performance in synchronization time and the encryption performance based on a chaotic complex system. The speed of synchronization control time is also a key factor affecting communication security and quality.

The types of synchronization time include asymptotic time synchronization, exponential synchronization, finite-time synchronization, fixed-time synchronization, and predefined-time synchronization. Asymptotic time synchronization and exponential synchronization are types of infinite time synchronization, but the convergence rate of exponential time is slightly faster than that of asymptotic time. However, finite-time stability has more practical significance in secure communication. Finite time was introduced in 1961 (Dorato, 1961), which has a much faster convergence time. In secure communication, compared with asymptotic synchronization and exponential synchronization, the finite-time synchronization technique enables us to recover the transmitted signals in a setting time, which improves the efficiency and the confidentiality greatly. The concept of fixed-time stability was proposed by Polyakov (2012), and the criteria for determining whether the system can achieve fixed-time stability were also attained. Fixed-time stability is a special kind of finite-time stability. It is different from general finite-time stability in that its stability time has a definite upper bound, and the settling upper bound time is not dependent on the initial value of the system, but can be calculated by the system parameters and controller parameters. Therefore, the research of fixed-time is still fascinating to many researchers, who have committed to exploring the smaller conservative of the fixed-time upper bound value. Unfortunately, the control issues of fixed-time algorithms are still challenges related to convergence time estimation. The main drawback is that convergence time is not explicit and controllable. In order to overcome the problems presented above, a new stability with a tunable parameter is proposed based on fixed-time stability, which is defined as predefined-time stability (Sanchez-Torres et al., 2014).

Tao and Chua (1997) attained the general stabilization of chaotic systems, and it was applied to secure communication. Then, many kinds of synchronization of more complex systems were also studied, which can also be applied to secure communication schemes. Many previous studies have made important considerations to the convergence of synchronization time. Therefore, during a period of time, many researchers have found many excellent results in the aspect of finite-time and fixed-time stabilities of coupled neural networks. Yang et al. (2015) and Yang and Lu (2016) focused on the finite-time synchronization of coupled neural networks and gave some novel conclusions. Some effective guiding conclusions are given in Lv et al. (2019) and Zhang et al. (2020). Since the existence of memristor was confirmed by the HP laboratory in 2008 (Strukov et al., 2008), many scholars introduced memristor in the study of neural networks, which can more truly simulate the memory characteristics of biological neural networks. Guo and Gao (2014), Wang et al. (2016), Chen et al. (2018), Zhang and Qi (2021), and Peng et al. (2020) considered the asymptotic time synchronization of CMNN and gave full consideration to various cases of random disturbances. Various results about exponential synchronization of CMNN (Wang and Shen, 2014; Bao et al., 2016; Feng et al., 2016; Guo et al., 2018; Chen et al., 2021) were given, and Guo et al. (2018) also considered multiple coupled terms. Especially, with the requirement of the development of converge speed, these results about finite/fixed time synchronization of CMNN are discussed (Li et al., 2019; Lu et al., 2019; Yang et al., 2019; Huang et al., 2020; Gong et al., 2021). Since the concept of predefined-time stability was proposed in 2014 (Sanchez-Torres et al., 2014), predefined-time stability in dynamical systems has been studied (Juan et al., 2018;), and some theorems of predefined-time stability have been obtained. Recently, Anguiano-Gijon et al. (2019), Lin (2021b), Assali (2021), Lin et al. (2021), and Muoz-Vazquez et al. (2021) went further and discussed the predefined-time synchronization of two chaotic systems, one of them obtained the projective synchronization criterion about predefined-time (Lin et al., 2021), and another two of them considered chaotic systems of fractional-order forms (Lin et al., 2021; Muoz-Vazquez et al., 2021). In 2020, Lin defined the novel criterion of predefined-time synchronization in different kinds of neural networks, including a chaos neural network and memristive neural network (Lin et al., 2020; Lin, 2021a).

For nearly 2 years, many effective conclusions were given in the research of synchronous control in a coupled memristive neural network. Zhou et al. (2020) constructed a novel synchronization about weighted sum synchronization for CMNN. Chen et al. (2021) and Feng et al. (2021) obtained some results in exponential and fixed-time synchronization of CMNN. Bao et al. (2021) completed further research on prescribed-time synchronization in CMNN. But, few papers fully take into account coupled topology and multi-links performance to explore the predefined-time stability of the systems, and give some application on image encryption and decryption schemes. The detailed contents of multi-links performance of complex networks are referred to by Zhao et al. (2015) and Zhao et al. (2016).

Motivated by the above discussions, multi-links performance, coupled forms, and synchronization time of systems are taken fully into account in this paper. We investigate the predefined-time synchronization of MCMNN and design an efficient secure communication scheme based on predefined-time stability. The contributions of this paper are given as follows: Firstly, we overcome the complexity of factor interaction including multi-links performance and coupled forms, dealing with some parameter mismatches and complex topological structure problems, the network model is more general; secondly, the predefined-time synchronization issue of drive-response MCMNN is first studied based on the feedback controller and give a new predefined-time stability theorem. The predefined-time is a case of fixed-time, the bound time can be explicitly tuned; thirdly, the secure communication scheme is designed based on predefined-time synchronization of drive-response MCMNN. The preset synchronization time can be used as an important common key, the plaintext signal can be recovered after the settling time; finally, numerical simulations are given to verify the effectiveness of our theoretical results and the feasibility of the secure communication scheme. Therefore, our work aims to fill some gaps in the research of coupled memristive neural networks and predefined-time synchronization.

The paper is organized as follows. In section 2, the model of MCMNN and preliminaries are explained. In section 3, the predefined-time synchronization theorem and corollaries are respectively shown. In section 4, we designed the security communication scheme based on predefined-time synchronization of MCMNN. The numerical example of predefined-time stability and application example of secure communication scheme are given to show the effectiveness of our theoretical results in section 5. Finally, the conclusion and prospects are given in section 6.

## 2. Network Model and Preliminaries

In the paper, according to the property of multi-links coupled topology, we consider a model of MCMNN as follows:


(1) $$\begin{array}{l} \begin{array}{c} {ẋ}_{ik}\left(t\right)=-c_{k}x_{ik}\left(t\right)+\overset{n}{\underset{q=1}{\sum }}a_{kq}\left(x_{ik}\left(t\right)\right){ḡ}_{q}\left(x_{ik}\left(t\right)\right)\\ \;+\overset{n}{\underset{q=1}{\sum }}b_{kq}\left(x_{ik}\left(t-\tau_{0}\right)\right)g_{q}\left(x_{ik}\left(t-\tau_{0}\right)\right)+\sigma \overset{N}{\underset{j=1}{\sum }}w_{ij}^{0}\Gamma x_{jk}\left(t\right)\\ \;+\sigma \overset{m}{\underset{l=1}{\sum }}\overset{N}{\underset{j=1}{\sum }}w_{ij}^{l}\Gamma x_{jk}\left(t-\tau_{l}\right)+I_{i}\left(t\right), \end{array} \end{array}$$

where $x_{i}={\left(x_{i1},x_{i2},...,x_{in}\right)}^{T}\in R^{n},i=1,2,...N$ is the state vector of the *i*th node; *C* = *diag*(*c*_1_, *c*_2_, …, *c*_*n*_) is a positive matrix and denotes the decay rates to the *i*th neuron. $ḡ\left(x_{i}\left(t\right)\right)=\left({ḡ}_{1}\left(x_{i1}\left(t\right)\right),{ḡ}_{2}\left(x_{i}\left(t\right)\right),...,{ḡ}_{n}\left(x_{in}\left(t\right)\right)\right)\in R^{n}$ and $g\left(x_{i}\left(t-\tau_{0}\right)\right)=\left(g_{1}\left(x_{i1}\left(t-\tau_{0}\right)\right),g_{2}\left(x_{i2}\left(t-\tau_{0}\right)\right),...,g_{n}\left(x_{in}\left(t-\tau_{0}\right)\right)\right)\in R^{n}$ are the discontinuous feedback functions, τ_0_ is time delay, σ represents the coupling strength, and Γ = *diag*(γ_1_, γ_2_, …, γ_*n*_) > 0 is the inner coupling matrix between each pair of nodes. $W_{0}={\left(w_{ij}^{0}\right)}_{N\times N},W_{l}={\left(w_{ij}^{l}\right)}_{N\times N},l=1,...,m$ represents the outer coupling configuration matrix of MNN, which is the different sub-network's Laplacian matrices, τ_*l*_(*l* = 1…, *m*) > 0 denote different time-delays in the sub-networks. If nodes *i* and *j* are linked by an edge, then $w_{ij}^{l}=w_{ji}^{l}>0\left(i\neq j\right)$, otherwise, $w_{ij}^{l}=w_{ji}^{l}=0$, and the diagonal elements of matrix *W*_*l*_ are defined as $w_{ii}^{l}=-\overset{N}{\underset{j=1,j\neq i}{\sum }}w_{ij}^{l}$. If there are no isolated nodes in the network, then all of the matrix *W*_*l*_(*l* = 0, 1, …, *m*) is an irreducible real symmetric matrix. $I\left(t\right)={\left(I_{1}\left(t\right),I_{2}\left(t\right),...,I_{N}\left(t\right)\right)}^{T}\in R^{n}$ is the external input.

The parameters *a*_*kq*_(*x*_*ik*_(*t*)) and *b*_*kq*_(*x*_*ik*_(*t*−τ_0_)) denote the non-delayed and delayed memristor-based synaptic connection weights, respectively. They can be described as follows:


(2) $$a_{kq}(x_{ik}(t))=\{\begin{array}{l} {\hat{a}}_{kq},|x_{ik}(t)|\leq T_{i},\\ {\overset{ˇ}{a}}_{kq},|x_{ik}(t)|>T_{i}, \end{array}$$

(3) $$b_{kq}(x_{ik}(t-\tau_{0}))=\{\begin{array}{l} {\hat{b}}_{kq},|x_{ik}(t-\tau_{0})|\leq T_{i},\\ {\overset{ˇ}{b}}_{kq},|x_{ik}(t-\tau_{0})|>T_{i}, \end{array}$$

where the switching jumps *T*_*i*_ > 0, â_*kq*_, ǎ_*kq*_, ${\hat{b}}_{kq}$, ${\hat{b}}_{kq}$, *k, q* = 1, 2, …, *n*, are all constants.

If Equation (1) denotes the drive system, the corresponding response system with a control input can be characterized by:


(4) $$\begin{array}{l} \begin{array}{c} {ẏ}_{ik}\left(t\right)=-c_{k}y_{ik}\left(t\right)+\overset{n}{\underset{q=1}{\sum }}a_{kq}\left(y_{ik}\left(t\right)\right){ḡ}_{q}\left(y_{ik}\left(t\right)\right)\\ \;+\overset{n}{\underset{q=1}{\sum }}b_{kq}\left(y_{ik}\left(t-\tau_{0}\right)\right)g_{q}\left(y_{ik}\left(t-\tau_{0}\right)\right)+\sigma \overset{N}{\underset{j=1}{\sum }}w_{ij}^{0}\Gamma y_{jk}\left(t\right)\\ \;+\sigma \overset{m}{\underset{l=1}{\sum }}\overset{N}{\underset{j=1}{\sum }}w_{ij}^{l}\Gamma y_{jk}\left(t-\tau_{l}\right)+I_{i}\left(t\right)+u_{ik}\left(t\right), \end{array} \end{array}$$

where *i, j* = 1, 2, …, *N*, $y_{i}={\left(y_{i1},y_{i2},...,y_{in}\right)}^{T}\in R^{n}$ is the state vector of the *i*th node of the response network and *u*_*ik*_(*t*) is the controller for node *i*. The remaining parameters of Equation (4) have the same meanings as those in Equation (1).

**Definition 1**. Filippov (1960) For a differential system: ẋ(*t*) = *f*(*t, x*), where *f*(*t, x*) is discontinuous in *x*(*t*), and *x*(*t*) is a solution of the differential system on [*t*_0_, *t*_1_] in Filippov's sense, if *x*(*t*) is absolutely continuous on any compact interval [*t*_0_, *t*_1_], for almost all *t* ∈ [*t*_0_, *t*_1_] such that


$$ẋ=K_{F}\left[f\right]\left(t,x\right),$$

where


$$K_{F}\left[f\right]\left(t,x\right)=\underset{\delta >0}{⋂}\underset{\mu \left(N\right)=0}{⋂}\bar{co}\left[f\left(B\left(x,\delta \right)\backslash N\right),t\right],$$

where $\bar{co}\left[\cdot \right]$ is the convex closure hull of a set, *B*(*x*, δ) = {*y*:||*y* − *x*|| ≤ δ} is the ball of center *x* and radius δ, the intersection is takes over all sets *N* of measure zero and over all δ > 0, and μ(*N*) is the Lebesgue measure of set *N*.

**Definition 2**. Polyakov (2012) considering the nonlinear system $\dot{v}=f\left(v,r\right)$ is said to suggest global fixed-time stability, if it has global finite-time stability and the settling time function *T*_max_ is bounded and independent of the initial conditions, i.e., there exists *T*_max_ > 0 such that


$$T\left(v\right)\leq T_{\max},\forall v_{0}\in R^{n}.$$

**Definition 3**. Sanchez-Torres et al. (2014) The drive-response systems (1) and (4) are said to achieve the predefined-time synchronization if there exists *T*_*v*_ in the case of fixed-time synchronization and if the settling time function $T:R^{n}\to R_{+}$ is such that


$$T\left(x_{0}\right)\leq T_{v},\forall x_{0}\in R^{n}.$$

**Assumption 1**. For the neuron activation functions ḡ_*q*_(·), *g*_*q*_(·), there exist Lipschitz constants ḡ_*q*_ and *g*_*q*_ > 0 satisfying the following Lipschitz conditions:


$$\begin{array}{l} ||{ḡ}_{q}\left(y\right)-{ḡ}_{q}\left(x\right)||\leq {\bar{f}}_{q}||\left(y-x\right)||,\\ ||g_{q}\left(y\right)-g_{q}\left(x\right)||\leq f_{q}||\left(y-x\right)||,x,y\in R. \end{array}$$

**Assumption 2**. The neuron activation functions are bounded functions, there exists a real number $M_{q},{\bar{M}}_{q}$ for any *g*_*q*_(*x*), ḡ_*q*_(*y*) such that $g_{q}\left(x\right)\leq M_{q},{ḡ}_{q}\left(y\right)\leq {\bar{M}}_{q}$.

**Lemma 1**. Hu et al. (2017) If there exists a regular, positive definite, and radially unbounded function $V\left(t\right):R^{n}\to R_{+}$ and constants *a* > 0, *b* > 0, η > 1 meet


$$\dot{V}\left(t\right)\leq -\left(aV^{\eta }\left(t\right)+b\right),t\in R^{n}\backslash 0,$$

the ∀*t* ≥ *T*_*max*_ of the origin system *V*(*t*) is fixed-time stability, and the upper-bounded settling time *T*_max_ is estimated by


$$T_{\max}=\frac{\eta }{b\left(\eta -1\right)}{\left(\frac{b}{a}\right)}^{\frac{1}{\eta }}.$$

**Lemma 2**. Khalil and Grizzle (2002) Let *a*_1_, *a*_2_, …, *a*_*N*_, η > 1, then the following inequality holds


$$\overset{N}{\underset{i=1}{\sum }}a_{i}^{\eta }\geq N^{1-\eta }(\overset{N}{\underset{i=1}{\sum }}a_{i}{)}^{\eta }.$$

**Remark 1**. The algebraic inequality of Lemma 2 is used in many studies to determine stability, such as Yang and Daniel (2016) and Yang et al. (2017), which focused on the research of exponential synchronization and finite-time synchronization in memristive neural networks without coupled links. Next, we extend the coupled memristive neural network model to explore predefined-time synchronization, in which the synchronization time can be adjusted in the controller.

**Lemma 3**. If there exists a regular, positive definite, and radially unbounded function $V\left(t\right):R^{n}\to R_{+}$ and constants *a* > 0, *b* > 0, η > 1 are satisfied


$$\dot{V}\left(t\right)\leq -\frac{D_{v}}{T_{v}}\left(aV^{\eta }\left(t\right)+b\right),t\in R^{n}\backslash 0,$$

where *T*_*v*_ is a user-defined parameter and


$$D_{v}=a^{-\frac{1}{\eta }}\cdot \frac{2^{\left(\eta -1\right)}}{\eta -1}\cdot b^{\frac{1-\eta }{\eta }}.$$

Then, for ∀*t* > *T*_*v*_, we have *V*(*t*) = 0. The origin system can achieve predefined-time synchronization.Proof: By Lemma 2, we have


$$\begin{array}{l} aV^{\eta }\left(t\right)+b={\left(a^{\frac{1}{\eta }}V\left(t\right)\right)}^{\eta }+{\left(b^{\frac{1}{\eta }}\right)}^{\eta }\\ \;\geq 2^{1-\eta }{\left(a^{\frac{1}{\eta }}V\left(t\right)+b^{\frac{1}{\eta }}\right)}^{\eta }. \end{array}$$

Then


$$\begin{array}{l} \begin{array}{c} T\left(x_{0}\right)\leq -\int_{V\left(x_{0}\right)}^{0}\frac{T_{v}}{D_{v}}\cdot \frac{1}{aV^{\eta }\left(t\right)+b}dV,\\ \;=-\int_{V\left(x_{0}\right)}^{0}\frac{T_{v}}{D_{v}}\cdot \frac{1}{{\left(a^{\frac{1}{\eta }}V\left(t\right)\right)}^{\eta }+{\left(b^{\frac{1}{\eta }}\right)}^{\eta }}dV,\\ \;\leq -\int_{V\left(x_{0}\right)}^{0}\frac{T_{v}}{D_{v}}\cdot \frac{1}{2^{1-\eta }{\left(a^{\frac{1}{\eta }}V\left(t\right)+b^{\frac{1}{\eta }}\right)}^{\eta }}dV,\\ \;=\frac{T_{v}}{D_{v}}\cdot 2^{\eta -1}\cdot \int_{0}^{V\left(x_{0}\right)}\frac{1}{{\left(a^{\frac{1}{\eta }}V\left(t\right)+b^{\frac{1}{\eta }}\right)}^{\eta }}dV,\\ \;=\frac{T_{v}}{D_{v}}\cdot \frac{2^{\eta -1}}{a^{\frac{1}{\eta }}\left(1-\eta \right)}\cdot {\left(a^{\frac{1}{\eta }}V\left(t\right)+b^{\frac{1}{\eta }}\right)}^{1-\eta }dV{|}_{0}^{V\left(x_{0}\right)},\\ \;=\frac{T_{v}}{D_{v}}\cdot \frac{2^{\eta -1}}{a^{\frac{1}{\eta }}\left(1-\eta \right)}\cdot \left({\left(a^{\frac{1}{\eta }}V\left(x_{0}\right)+b^{\frac{1}{\eta }}\right)}^{1-\eta }-b^{\frac{1-\eta }{\eta }}\right),\\ \;=\frac{T_{v}}{D_{v}}\cdot \frac{2^{\eta -1}}{a^{\frac{1}{\eta }}\left(\eta -1\right)}\cdot \left(b^{\frac{1-\eta }{\eta }}-{\left(a^{\frac{1}{\eta }}V\left(x_{0}\right)+b^{\frac{1}{\eta }}\right)}^{1-\eta }\right),\\ \;=\frac{T_{v}}{D_{v}}\cdot \frac{2^{\eta -1}}{a^{\frac{1}{\eta }}\left(\eta -1\right)}\cdot \left(b^{\frac{1-\eta }{\eta }}-\frac{1}{{\left(a^{\frac{1}{\eta }}V\left(x_{0}\right)+b^{\frac{1}{\eta }}\right)}^{\eta -1}}\right). \end{array} \end{array}$$

If *V*(*x*_0_) = 0, then *T*(*x*_0_) = 0. If *V*(*x*_0_) → ∞, then $\frac{1}{{\left(a^{\frac{1}{\eta }}V\left(x_{0}\right)+b^{\frac{1}{\eta }}\right)}^{\eta -1}}\to 0$, thus we have


$$\begin{array}{l} T\left(x_{0}\right)\leq \frac{T_{v}}{D_{v}}\cdot \frac{2^{\eta -1}}{a^{\frac{1}{\eta }}\left(\eta -1\right)}\cdot \left(b^{\frac{1-\eta }{\eta }}-\frac{1}{{\left(a^{\frac{1}{\eta }}V\left(x_{0}\right)+b^{\frac{1}{\eta }}\right)}^{\eta -1}}\right),\\ \;\leq \frac{T_{v}}{D_{v}}\cdot a^{-\frac{1}{\eta }}\cdot \frac{2^{\eta -1}}{\eta -1}\cdot b^{\frac{1-\eta }{\eta }}=T_{v}. \end{array}$$

The proof is completed.

Denote


$$\begin{array}{l} {\bar{a}}_{kq}=\max\{{\hat{a}}_{kq},{\overset{ˇ}{a}}_{kq}\},{\underline{a}}_{kq}=\min\{{\hat{a}}_{kq},{\overset{ˇ}{a}}_{kq}\},\\ {\bar{b}}_{kq}=\max\{{\hat{b}}_{kq},{\overset{ˇ}{b}}_{kq}\},{\underline{b}}_{kq}=\min\{{\hat{b}}_{kq},{\overset{ˇ}{b}}_{kq}\},\\ a_{kq}=\frac{1}{2}({\bar{a}}_{kq}+{\underline{a}}_{kq}),{\tilde{a}}_{kq}=\frac{1}{2}({\bar{a}}_{kq}-{\underline{a}}_{kq}),\\ b_{kq}=\frac{1}{2}({\bar{b}}_{kq}+{\underline{b}}_{kq}),{\tilde{b}}_{kq}=\frac{1}{2}({\bar{b}}_{kq}-{\underline{b}}_{kq}). \end{array}$$

Therefore, based on Definition 1 and the theory of differential inclusion, Equation (1) and Equation (4) can be written as


(5) $$\begin{array}{l} \begin{array}{c} {ẋ}_{ik}\left(t\right)\in -c_{k}x_{ik}\left(t\right)+\overset{n}{\underset{q=1}{\sum }}\left(a_{kq}+\bar{co}\left[-{ã}_{kq},{ã}_{kq}\right]\right){ḡ}_{q}\left(x_{ik}\left(t\right)\right)\\ \;+\overset{n}{\underset{q=1}{\sum }}\left(b_{kq}+\bar{co}\left[-{\tilde{b}}_{kq},{\tilde{b}}_{kq}\right]\right)g_{q}\left(x_{ik}\left(t-\tau_{0}\right)\right)\\ \;+\sigma \overset{N}{\underset{j=1}{\sum }}w_{ij}^{0}\gamma_{k}x_{jk}\left(t\right)\\ \;+\sigma \overset{m}{\underset{l=1}{\sum }}\overset{N}{\underset{j=1}{\sum }}w_{ij}^{l}\gamma_{k}x_{jk}\left(t-\tau_{l}\right)+I_{i}\left(t\right), \end{array} \end{array}$$

and


(6) $$\begin{array}{l} \begin{array}{c} {ẏ}_{ik}\left(t\right)\in -c_{k}y_{ik}\left(t\right)+\overset{n}{\underset{q=1}{\sum }}\left(a_{kq}+\bar{co}\left[-{ã}_{kq},{ã}_{kq}\right]\right){ḡ}_{q}\left(y_{ik}\left(t\right)\right)\\ \;+\overset{n}{\underset{q=1}{\sum }}\left(b_{kq}+\bar{co}\left[-{\tilde{b}}_{kq},{\tilde{b}}_{kq}\right]\right)g_{q}\left(y_{ik}\left(t-\tau_{0}\right)\right)\\ \;+\sigma \overset{N}{\underset{j=1}{\sum }}w_{ij}^{0}\gamma_{k}y_{jk}\left(t\right)\\ \;+\sigma \overset{m}{\underset{l=1}{\sum }}\overset{N}{\underset{j=1}{\sum }}w_{ij}^{l}\gamma_{k}y_{jk}\left(t-\tau_{l}\right)+I_{i}\left(t\right)+u_{ik}\left(t\right). \end{array} \end{array}$$

**Remark 2**. According to the state-dependence conditions of Equations (5) and (6), the variables $\bar{co}\left[-{ã}_{kq},{ã}_{kq}\right],\bar{co}\left[-{\tilde{b}}_{kq},{\tilde{b}}_{kq}\right]$ may not reach their maximum and minimum values at the same time. Therefore, we give the following four different measurable functions to represent interval information.

According to the measurable selection theorem (Xiao and Zeng, 2017), there exist measurable functions $\xi_{kq}^{1}\left(t\right),\xi_{kq}^{2}\left(t\right),\xi_{kq}^{3}\left(t\right),\xi_{kq}^{4}\left(t\right)\in \bar{co}\left[-1,1\right]$ such that


(7) $$\begin{array}{l} {\dot{x}}_{ik}(t)=-c_{k}x_{ik}(t)+\sum_{q=1}^{n}(a_{kq}+{\tilde{a}}_{kq}\xi_{kq}^{1}(t){\bar{g}}_{q}(x_{ik}(t))\\ \;+\sum_{q=1}^{n}(b_{kq}+{\tilde{b}}_{kq}\xi_{kq}^{3}(t)g_{q}(x_{ik}(t-\tau_{0}))+\sigma \sum_{j=1}^{N}w_{ij}^{0}\gamma_{k}x_{jk}(t)\\ \;+\sigma \sum_{l=1}^{m}\sum_{j=1}^{N}w_{ij}^{l}\gamma_{k}x_{jk}(t-\tau_{l})+I_{i}(t), \end{array}$$

and


(8) $$\begin{array}{l} {\dot{y}}_{ik}(t)=-c_{k}y_{ik}(t)+\sum_{q=1}^{n}(a_{kq}+{\tilde{a}}_{kq}\xi_{kq}^{2}(t){\bar{g}}_{q}(y_{ik}(t))\\ \;+\sum_{q=1}^{n}(b_{kq}+{\tilde{b}}_{kq}\xi_{kq}^{4}(t)g_{q}(y_{ik}(t-\tau_{0}))+\sigma \sum_{j=1}^{N}w_{ij}^{0}\gamma_{k}y_{jk}(t)\\ \;+\sigma \sum_{l=1}^{m}\sum_{j=1}^{N}w_{ij}^{l}\gamma_{k}y_{jk}(t-\tau_{l})+I_{i}(t)+u_{ik}(t). \end{array}$$

Let *e*_*ik*_(*t*) = *y*_*ik*_(*t*)−*x*_*ik*_(*t*), the corresponding error system is given as follows:


(9) $$\begin{array}{l} {\dot{e}}_{ik}(t)=-c_{k}e_{ik}(t)+\sum_{q=1}^{n}a_{kq}({\bar{g}}_{q}(y_{ik}(t))-{\bar{g}}_{q}(x_{ik}(t)))\\ \;+\sum_{q=1}^{n}{\tilde{a}}_{kq}\xi_{kq}^{2}(t)({\bar{g}}_{q}(y_{ik}(t))-{\bar{g}}_{q}(x_{ik}(t)))\\ \;+\sum_{q=1}^{n}{\tilde{a}}_{kq}(\xi_{kq}^{2}(t)-\xi_{kq}^{1}(t)){\bar{g}}_{q}(x_{ik}(t))\\ \;+\sum_{q=1}^{n}b_{kq}(g_{q}(y_{ik}(t-\tau_{0}))-g_{q}(x_{ik}(t-\tau_{0}))\\ \;+\sum_{q=1}^{n}{\tilde{b}}_{kq}\xi_{kq}^{4}(t)({\bar{g}}_{q}(y_{ik}(t-\tau_{0}))-{\bar{g}}_{q}(x_{ik}(t-\tau_{0})))\\ \;+\sum_{q=1}^{n}{\tilde{b}}_{kq}(\xi_{kq}^{4}(t)-\xi_{kq}^{3}(t)){\bar{g}}_{q}(x_{ik}(t-\tau_{0}))\\ \;+\sigma \sum_{j=1}^{N}w_{ij}^{0}\gamma_{k}e_{jk}(t)+\sigma \sum_{l=1}^{m}\sum_{j=1}^{N}w_{ij}^{l}\gamma_{k}e_{jk}(t-\tau_{l})+u_{ik}(t). \end{array}$$

**Remark 3**. Based on Definition 3, the issues of predefined-time synchronization between drive system (1) and response system (4) are transformed into the issues of predefined-time stability of error system (9).

## 3. Predefined-Time Synchronization for MCMNN

In order to guarantee the predefined-time synchronization of drive-response systems, the controller is designed as follows:


(10) $$\begin{array}{l} u_{ik}(t)=-\alpha_{i}e_{ik}(t)-sign(e_{ik}(t))(\beta_{i}+\sum_{l=0}^{m}r_{i}|e_{ik}(t-\tau_{l})|)\\ \;+\frac{D_{v}}{T_{v}}\delta_{i}|e_{ik}(t){|}^{\eta }), \end{array}$$

where *i* = 1, 2, …, *N, k* = 1, 2, .., *n*. α_*i*_, β_*i*_, *r*_*i*_, δ_*i*_, η ≥ 0. *T*_*v*_ is the tunable predefined time, *D*_*v*_ is a positive constant given by other parameters. And sign(*x*) is the sign function which is defined as follows:


$$sign(x)=\{\begin{array}{cc} -1, & ifx<0,\\ 0, & ifx=0,\\ 1, & ifx>0. \end{array}$$

**Remark 4**. Designed controller (10) is discontinuous. To ensure the existence of the solutions of error system (11), the Dini derivative is used to ensure continuity at the breakpoint.

According to Assumptions 1 and 2, combined with designed controller (10), we obtain


$$\begin{array}{l} {\dot{e}}_{ik}(t)\leq -c_{k}e_{ik}(t)+\sum_{q=1}^{n}|a_{kq}|{\bar{f}}_{q}|e_{ik}(t)|+\sum_{q=1}^{n}{\tilde{a}}_{kq}\xi_{kq}^{2}(t){\bar{f}}_{q}|e_{ik}(t)|\\ \;+2\sum_{q=1}^{n}{\tilde{a}}_{kq}{\bar{M}}_{q}+\sum_{q=1}^{n}|b_{kq}|f_{q}|e_{ik}(t-\tau_{0})|\\ \;+\sum_{q=1}^{n}{\tilde{b}}_{kq}\xi_{kq}^{4}(t)f_{q}|e_{ik}(t-\tau_{0})|+2\sum_{q=1}^{n}{\tilde{b}}_{kq}M_{q}\\ \;+\sigma \sum_{j=1}^{N}w_{ij}^{0}\gamma_{k}e_{jk}(t)+\sigma \sum_{l=1}^{m}\sum_{j=1}^{N}w_{ij}^{l}\gamma_{k}e_{jk}(t-\tau_{l})\\ \;-\alpha_{i}e_{ik}(t)-sign(e_{ik}(t)(\beta_{i}+\sum_{l=0}^{m}r_{i}e_{ik}(t-\tau_{l})\\ \;+\frac{D_{v}}{T_{v}}\delta_{i}|e_{ik}(t){|}^{\eta }). \end{array}$$

Based on designed controller (10) applied on the response system, a theorem is presented to achieve the predefined-time synchronization for MCMNN.

Denote $\gamma_{\max}=\underset{1\leq k\leq n}{\max}\left(\gamma_{k}\right)$, α = *diag*(α_1_, α_2_, …, α_*N*_), β = *diag*(β_1_, β_2_, …, β_*N*_),

*r* = *diag*(*r*_1_, *r*_2_, …, *r*_*N*_), $W_{l}={\left(w_{ij}^{l}\right)}_{N\times N},l=1,2,...m$.

**Theorem 1**. Under Assumptions 1 and 2, for a predefined-time *T*_*v*_ > 0 and controller (10), error system (9) can achieve predefined-time stability if


$$\left\{ \begin{array}{l} \phi_{1}I_{N}-\alpha +\sigma \gamma_{max}W_{0}\leq 0,\\ \phi_{2}I_{N}-r\leq 0,\\ \sigma \gamma_{max}\sum_{l=1}^{m}W_{l}-mr\leq 0,\\ \phi_{3}I_{N}-\beta \leq 0. \end{array}\right.$$

where


$$\left\{ \begin{array}{l} \phi_{1}=\underset{1\leq k\leq n}{\max}\{-c_{k}+\sum_{q=1}^{n}\left(|a_{kq}|+{\tilde{a}}_{kq}\right){\bar{f}}_{q}\},\\ \phi_{2}=\underset{1\leq k\leq n}{\max}\{\sum_{q=1}^{n}\left(|b_{kq}|+{\tilde{b}}_{kq}\right)f_{q}\},\\ \phi_{3}=\underset{1\leq k\leq n}{\max}\{2\sum_{q=1}^{n}\left({\tilde{a}}_{kq}{\bar{M}}_{q}+{\tilde{B}}_{kq}M_{q}\right). \end{array}\right.$$

Proof: We construct a Lyapunov function as follows:


$$\begin{array}{l} V\left(e\left(t\right)\right)=\overset{N}{\underset{i=1}{\sum }}||e_{i}\left(t\right)|{|}_{1}=\overset{N}{\underset{i=1}{\sum }}\overset{n}{\underset{k=1}{\sum }}|e_{ik}\left(t\right)|. \end{array}$$

When *e*(*t*) = 0, *V*(*e*(*t*)) = 0 and the derivative of *V*(*e*(*t*)) is 0. Then, the derivative of *V*(*e*(*t*)) is along the trajectories of *e*(*t*) with *e*(*t*) ≠ 0. We have


$$\begin{array}{l} \dot{V}(e(t))=\sum_{i=1}^{N}\sum_{k=1}^{n}sign(e_{ik}(t)){\dot{e}}_{ik}(t),\\ \;\leq \sum_{i=1}^{N}\sum_{k=1}^{n}-c_{k}|e_{ik}(t)|+\sum_{i=1}^{N}\sum_{k=1}^{n}\sum_{q=1}^{n}\left(|a_{kq}|+{\tilde{a}}_{kq}\right){\bar{f}}_{q}|e_{ik}(t)|)\\ \;+\sum_{i=1}^{N}\sum_{k=1}^{n}\sum_{q=1}^{n}\left(|b_{kq}|+{\tilde{b}}_{kq}\right)f_{q}|e_{ik}(t-\tau_{0})|)\\ \;+2\sum_{i=1}^{N}\sum_{k=1}^{n}\sum_{q=1}^{n}\left({\tilde{a}}_{kq}{\bar{M}}_{q}+{\tilde{b}}_{kq}M_{q}\right)\\ \;+\sigma \sum_{i=1}^{N}\sum_{j=1}^{N}\sum_{k=1}^{n}w_{ji}^{0}\gamma_{k}|e_{ik}(t)|\\ \;+\sigma \sum_{i=1}^{N}\sum_{j=1}^{N}\sum_{k=1}^{n}\sum_{l=1}^{m}w_{ji}^{l}\gamma_{k}|eik(t-\tau_{l})|\\ \;-\sum_{i=1}^{N}\sum_{k=1}^{n}\alpha_{i}|e_{ik}(t)|-\sum_{i=1}^{N}\sum_{k=1}^{n}\beta_{i}\\ \;-\sum_{i=1}^{N}\sum_{k=1}^{n}\sum_{l=0}^{m}r_{i}|e_{ik}(t-\tau_{l})|\\ \;-\frac{D_{v}}{T_{v}}\sum_{i=1}^{N}\sum_{k=1}^{n}\delta_{i}|e_{ik}(t){|}^{\eta }). \end{array}$$

According to the analysis above, we can obtain that


$$\begin{array}{l} \dot{V}(e(t))\leq \sum_{i=1}^{N}\sum_{k=1}^{n}-c_{k}|e_{ik}(t)|+\sum_{q=1}^{n}\left(a_{kq}+{\tilde{a}}_{kq}\right){\bar{f}}_{q}\\ \;+\sigma \sum_{j=1}^{N}w_{ji}^{0}\gamma_{\max}-\alpha_{i}]|e_{ik}(t)|\\ \;+\sum_{i=1}^{N}\sum_{k=1}^{n}[\sum_{q=1}^{n}\left(|b_{kq}|+{\tilde{b}}_{kq}\right)f_{q}-r_{i}]|e_{ik}(t-\tau_{0})|\\ \;+\sum_{i=1}^{N}\sum_{k=1}^{n}\sum_{l=1}^{m}\left(\sigma \sum_{j=1}^{N}w_{ji}^{l}\gamma_{\max}-r_{i})|e_{ik}(t-\tau_{l}\right)|\\ \;-\sum_{i=1}^{N}\sum_{k=1}^{n}\left[\beta_{i}-2\sum_{q=1}^{n}\left({\tilde{a}}_{kq}{\bar{M}}_{q}+{\tilde{b}}_{kq}M_{q}\right)\right]\\ \;-\frac{D_{v}}{T_{v}}\sum_{i=1}^{N}\sum_{k=1}^{n}\delta_{i}|e_{ik}(t){|}^{\eta }),\\ \;\leq -\frac{D_{v}}{T_{v}}\sum_{i=1}^{N}\sum_{k=1}^{n}\delta_{i}|e_{ik}(t){|}^{\eta }\\ \;-\sum_{i=1}^{N}\sum_{k=1}^{n}\left[\beta_{i}-2\sum_{q=1}^{n}\left({\tilde{a}}_{kq}{\bar{M}}_{q}+{\tilde{b}}_{kq}M_{q}\right)\right]. \end{array}$$

Let $\lambda =\underset{1\leq i\leq N}{\min}\left\{\delta_{i}\right\}$, $\underset{1\leq i\leq N}{\min}\left(\beta_{i}-\phi_{3}\right)\geq \frac{D_{v}}{T_{v}}b$, and by Lemma 2, we obtain


$$\begin{array}{l} -\frac{D_{v}}{T_{v}}\overset{N}{\underset{i=1}{\sum }}\overset{n}{\underset{k=1}{\sum }}\delta_{i}|e_{ik}\left(t\right){|}^{\eta }\leq -\frac{D_{v}}{T_{v}}\lambda n^{1-\eta }{\left(V\left(e\left(t\right)\right)\right)}^{\eta },\\ -\overset{N}{\underset{i=1}{\sum }}\overset{n}{\underset{k=1}{\sum }}\left[\beta_{i}-2\overset{n}{\underset{q=1}{\sum }}\left({ã}_{kq}{\bar{M}}_{q}+{\tilde{b}}_{kq}M_{q}\right)\right]\leq \frac{D_{v}}{T_{v}}b. \end{array}$$

According to the above proof and *a* = λ*n*^1−η^, we have:


$$\begin{array}{l} \begin{array}{c} \dot{V}\left(e\left(t\right)\right)\leq -\frac{D_{v}}{T_{v}}\lambda n^{1-\eta }{\left(V\left(e\left(t\right)\right)\right)}^{\eta }-\frac{D_{v}}{T_{v}}b,\\ =-\frac{D_{v}}{T_{v}}\left(a{\left(V\left(e\left(t\right)\right)\right)}^{\eta }+b\right), \end{array} \end{array}$$

where


$$D_{v}=a^{-\frac{1}{\eta }}\cdot \frac{2^{\left(\eta -1\right)}}{\eta -1}\cdot b^{\frac{1-\eta }{\eta }}.$$

The proof of Theorem 1 is completed.

**Remark 5**. The predefined-time stability is a spacial case of fixed-time stability. Therefore, after removing the tuning parameters *T*_*v*_ and *D*_*v*_, the error system is said to achieve fixed-time stability, and the upper bound of settling time can be indicated as *T*_*max*_ = *D*_*v*_ based on Definition 2 and Lemma 2. Therefore, error system (9) can achieve fixed-time stability based on the above controller (10).

The results of Theorem 1 can also easily extend to the general single coupled memristive neural network which does not include multi-links items. The drive-response systems are given as


(11) $$\left\{ \begin{array}{l} {\dot{x}}_{ik}(t)=-c_{k}x_{ik}(t)+\sum_{q=1}^{n}a_{kq}(x_{ik}(t)){\bar{g}}_{q}(x_{ik}(t))\\ \;+\sum_{q=1}^{n}b_{kq}(x_{ik}(t-\tau_{0}))g_{q}(x_{ik}(t-\tau_{0}))\\ \;+\sigma \sum_{j=1}^{N}w_{ij}^{0}\Gamma x_{jk}(t)+I_{i},\\ {\dot{y}}_{ik}(t)=-c_{k}y_{ik}(t)+\sum_{q=1}^{n}a_{kq}(y_{ik}(t)){\bar{g}}_{q}(y_{ik}(t))\\ \;+\sum_{q=1}^{n}b_{kq}(y_{ik}(t-\tau_{0}))g_{q}(y_{ik}(t-\tau_{0}))\\ \;+\sigma \sum_{j=1}^{N}w_{ij}^{0}\Gamma y_{jk}(t)+I_{i}+u_{ik}(t). \end{array}\right.$$

The controller is designed as follows:


(12) $$\begin{array}{l} u_{ik}=-\alpha_{i}e_{ik}\left(t\right)-sign\left(e_{ik}\left(t\right)\right)\left(\beta_{i}+r_{i}|e_{ik}\left(t-\tau_{0}\right)|+\frac{D_{v}}{T_{v}}\delta_{i}|e_{ik}{|}^{\eta }\right). \end{array}$$

According to drive-response system (12), the corresponding corollary is given as

**Corollary 1**. Under Assumptions 1 and 2 and controller (13), drive-response system (12) can achieve predefined-time synchronization if


$$\left\{ \begin{array}{l} \phi_{1}I_{N}-\alpha +\sigma \gamma_{max}W_{0}\leq 0,\\ \phi_{2}I_{N}-r\leq 0,\\ \phi_{3}I_{N}-\beta \leq 0. \end{array}\right.$$

The results of Theorem 1 can also further extend to the general memristive neural network which does not include coupled topology. The drive-response systems are given as


(13) $$\left\{ \begin{array}{l} {\dot{x}}_{ik}(t)=-c_{k}x_{ik}(t)+\sum_{q=1}^{n}a_{kq}(x_{ik}(t)){\bar{g}}_{q}(x_{ik}(t))\\ \;+\sum_{q=1}^{n}b_{kq}(x_{ik}(t-\tau_{0}))g_{q}(x_{ik}(t-\tau_{0}))+I_{i},\\ {\dot{y}}_{ik}(t)=-c_{k}y_{ik}(t)+\sum_{q=1}^{n}a_{kq}(y_{ik}(t)){\bar{g}}_{q}(y_{ik}(t))\\ \;+\sum_{q=1}^{n}b_{kq}(y_{ik}(t-\tau_{0}))g_{q}(y_{ik}(t-\tau_{0}))+I_{i}+u_{ik}(t). \end{array}\right.$$

According to drive-response system (14), we give the corresponding corollary as follows:

**Corollary 2**. Under Assumptions 1 and 2 and controller (13), drive-response system (14) can achieve predefined-time synchronization if,


$$\{\begin{array}{c} \phi_{1}I_{n}-\alpha \leq 0,\\ \phi_{2}I_{n}-r\leq 0,\\ \phi_{3}I_{n}-\beta \leq 0. \end{array}$$

## 4. Design of the Secure Communication Scheme

This section presents the secure communication scheme based on the predefined-time synchronous control of MCMNN, which comprises the following steps:

Step 1: The three-dimensional drive-response systems *x*_*i*_(*t*) and *y*_*i*_(*t*) are built, which together are the MCMNN;

Step 2: According to the drive-response systems, the synchronization error system *e*_*i*_(*t*) = *y*_*i*_(*t*) − *x*_*i*_(*t*) is established;

Step 3: The predefined-time stability theorem (Theorem 1) with low conservation is adopted;

Step 4: The appropriate predefined-time synchronization controller *u*_*i*_(*t*) is designed;

Step 5: A new predefined-time synchronization control theorem is given to realize the predefined-time stability of the error system, and the controllability of stability time is guaranteed via the tuning parameter *T*_*v*_;

Step 6: The implementation of the secure communication scheme:

**Sender:** The mixed signal generated by the plaintext signal and the prefixed random signal is introduced into the drive system of the coupled memristive neural network with multi-links, and the sender generates the encrypted signal by superimposing the drive system signal and mixed signal, and sends it to the receiver through the transmission channel.

The designed plaintext signals without any encryption are *m*_*i*_(*t*), *i* = 1, 2, 3.

The mixed signal by the plaintext signals and random signal are given as


$$M_{i}(t)=\left\{ \begin{array}{l} r_{i}(t),0\leq t<T_{v},\\ m_{i}(t-T_{v}),t\geq T_{v}. \end{array}\right.$$

where *i* = 1, 2, 3.

The encrypted signal by superimposing the drive system signal and mixed signal are *E*_*i*_(*t*) = *M*_*i*_(*t*) + *x*_*i*_(*t*).

**Remark 6**. The use of the random signal before the encrypted signal is to enhance the security of the transmit signals.

**Receiver:** The received transmitted signal, the known key, and public parameter information are introduced into the response system. The predefined-time stability theorem given by step 3 and the predefined-time synchronization of drive-response systems are realized under the synchronization controller. The receiver can decrypt the plaintext signal after setting the predefined synchronization time.

According to the predefined time *T*_*v*_, if *t* > *T*_*v*_, then *x*_*i*_(*t*) = *y*_*i*_(*t*). The receiver can decrypt the plaintext signal by the following formula:


$$\begin{array}{l} \begin{array}{c} m_{i}^{\prime }\left(t\right)=E_{i}\left(t+T_{v}\right)-y_{i}\left(t+T_{v}\right),\\ \;=M_{i}\left(t+T_{v}\right)+x_{i}\left(t+T_{v}\right)-y_{i}\left(t+T_{v}\right),\\ \;=M_{i}\left(t+T_{v}\right),\\ \;=m_{i}\left(t\right),t\geq 0. \end{array} \end{array}$$

**Note**: The sender and the receiver have a common key; all parameters of the drive system generated by the sender are public; after generating the drive system, the sender destroys the initial value of the system which cannot be disclosed.

The transmission signal and the recovery plaintext signal can improve the transmission efficiency and ensure the security of signal transmission in the secure communication scheme based on the predefined-time synchronous control of MCMNN. This scheme has the following advantages:

(1) We can use Simulink in MATLAB to build a three-dimensional MCMNN or program simulation in MATLAB to design a secure communication scheme based on predefined-time synchronous control of the drive-response systems, and the design scheme is flexible.

(2) In the secure communication scheme, the encrypted signal superimposed by the three-dimensional system is relatively complex and is not easy to crack.

(3) In the secure communication scheme, both the drive system and the response system contain three differential equations, and the secure communication is realized under the synchronous control of the drive-response system, which provides a new perspective for the research of secure communication.

(4) The scheme can preset the synchronization time according to the need, and it can predict the communication time more accurately and effectively, and improve the efficiency and security of the transmission information.

(5) The secure communication scheme has good expansibility and can be applied to the encrypted transmission of various images, videos, and other signals and the abnormal detection system of information.

## 5. Numerical Simulations

**Example 1:** Consider the following three-neuron CMNN with three-links as drive-response systems:


(14) $$\left\{ \begin{array}{l} {\dot{x}}_{ik}(t)=-c_{k}x_{ik}(t)+a_{kq}(x_{ik}(t))\bar{g}(x_{ik}(t))\\ \;+b_{kq}(x_{ik}(t-\tau_{0}))g(x_{ik}(t-\tau_{0}))\\ \;+\sigma \sum_{j=1}^{N}w_{ij}^{0}\Gamma x_{jk}(t)+\sigma \sum_{j=1}^{N}w_{ij}^{1}\Gamma x_{jk}(t-\tau_{1})\\ \;+\sigma \sum_{j=1}^{N}w_{ij}^{2}\Gamma x_{jk}(t-\tau_{2})+I_{i}(t),\\ {\dot{y}}_{ik}(t)=-c_{k}y_{ik}(t)+a_{kq}(y_{ik}(t))\bar{g}(y_{ik}(t))\\ \;+b_{kq}(y_{ik}(t-\tau_{0}))g(y_{ik}(t-\tau_{0}))\\ \;+\sigma \sum_{j=1}^{N}w_{ij}^{0}\Gamma y_{jk}(t)+\sigma \sum_{j=1}^{N}w_{ij}^{1}\Gamma y_{jk}(t-\tau_{1})\\ \;+\sigma \sum_{j=1}^{N}w_{ij}^{2}\Gamma y_{jk}(t-\tau_{2})+I_{i}(t)+u_{i}(t), \end{array}\right.$$

where *i* = 1, 2, …, 8, *c* = *diag*(5, 6, 7), *N* = 8, σ = 1, *and Γ* = *I*_3×3_. *I*_*i*_(*t*) can be omitted in the simulation. The active functions are $ḡ\left(x\right)=\frac{1}{2}|\left(\left|x+1|-|x-1\right|\right)|-1\;and\;g\left(x\right)=\frac{1}{4}\left(\left|x+1|-|x-1\right|\right)$. The time-delays are τ_0_ = 0.1, τ_1_ = 0.2, *and τ*_2_ = 0.4. The initial values of drive-response systems (14) and (15) are given by:


$$\begin{array}{l} x\left(0\right)=\left[5+i,1+3i,2+5i\right],y\left(0\right)=\left[2+4i,-2+4i,3+4i\right],\\ i=1,2,...,8. \end{array}$$

The weight parameters are given by


$$\begin{array}{l} a_{11}(x_{i1})=\{\begin{array}{l} \begin{array}{c} -0.8,|x_{i1}(t)|\leq 1,\\ -1,|x_{i1}(t)|>1, \end{array} \end{array}a_{12}(x_{i1})=\{\begin{array}{l} \begin{array}{c} 2.2,|x_{i1}(t)|\leq 1,\\ 2,|x_{i1}(t)|>1, \end{array} \end{array}\\ \;a_{13}(x_{i1})=\{\begin{array}{l} \begin{array}{c} 1.2,|x_{i1}(t)|\leq 1,\\ 1.8,|x_{i1}(t)|>1, \end{array} \end{array} \end{array}$$

$$\begin{array}{l} a_{21}(x_{i2})=\{\begin{array}{l} \begin{array}{c} 1,|x_{i2}(t)|\leq 1,\\ 0.8,|x_{i2}(t)|>1, \end{array} \end{array}a_{22}(x_{i2})=\{\begin{array}{l} \begin{array}{c} -1,|x_{i2}(t)|\leq 1,\\ -0.8,|x_{i2}(t)|>1. \end{array} \end{array}\\ \;a_{23}(x_{i2})=\{\begin{array}{l} \begin{array}{c} -2.4,|x_{i2}(t)|\leq 1,\\ -2,|x_{i2}(t)|>1, \end{array} \end{array} \end{array}$$

$$\begin{array}{l} a_{31}(x_{i3})=\{\begin{array}{l} \begin{array}{c} 0.2,|x_{i3}(t)|\leq 1,\\ 0.4,|x_{i3}(t)|>1, \end{array} \end{array}a_{32}(x_{i3})=\{\begin{array}{l} \begin{array}{c} -0.6,|x_{i3}(t)|\leq 1,\\ -0.4,|x_{i3}(t)|>1. \end{array} \end{array}\\ \;a_{33}(x_{i3})=\{\begin{array}{l} \begin{array}{c} -1.8,|x_{i3}(t)|\leq 1,\\ -1.2,|x_{i3}(t)|>1, \end{array} \end{array} \end{array}$$

$$\begin{array}{l} b_{11}(x_{i1})=\{\begin{array}{l} \begin{array}{c} -3.2,|x_{i1}(t-\tau_{0})|\leq 1,\\ -3,|x_{i1}(t-\tau_{0})|>1, \end{array} \end{array}b_{12}(x_{i1})=\{\begin{array}{l} \begin{array}{c} 0.2,|x_{i1}(t-\tau_{0})|\leq 1,\\ 0.4,|x_{i1}(t-\tau_{0})|>1, \end{array} \end{array}\\ \;b_{13}(x_{i1})=\{\begin{array}{l} \begin{array}{c} 1,|x_{i1}(t-\tau_{0})|\leq 1,\\ 1.5,|x_{i1}(t-\tau_{0})|>1, \end{array} \end{array} \end{array}$$

$$\begin{array}{l} b_{21}(x_{i2})=\{\begin{array}{l} \begin{array}{c} 0.4,|x_{i2}(t-\tau_{0})|\leq 1,\\ 0.2,|x_{i2}(t-\tau_{0})|>1, \end{array} \end{array}b_{22}(x_{i2})=\{\begin{array}{l} \begin{array}{c} -3.6,|x_{i2}(t-\tau_{0})|\leq 1,\\ -3.2,|x_{i2}(t-\tau_{0})|>1. \end{array} \end{array}\\ \;b_{23}(x_{i2})=\{\begin{array}{l} \begin{array}{c} 1.5,|x_{i2}(t-\tau_{0})|\leq 1,\\ 2.1,|x_{i2}(t-\tau_{0})|>1. \end{array} \end{array} \end{array}$$

$$\begin{array}{l} b_{31}(x_{i3})=\{\begin{array}{l} \begin{array}{c} 2.2,|x_{i3}(t-\tau_{0})|\leq 1,\\ 2.6,|x_{i3}(t-\tau_{0})|>1, \end{array} \end{array}b_{32}(x_{i3})=\{\begin{array}{l} \begin{array}{c} 3.2,|x_{i3}(t-\tau_{0})|\leq 1,\\ 2.8,|x_{i2}(t-\tau_{0})|>1. \end{array} \end{array}\\ \;b_{33}(x_{i3})=\{\begin{array}{l} \begin{array}{c} 2.6,|x_{i3}(t-\tau_{0})|\leq 1,\\ 2.4,|x_{i3}(t-\tau_{0})|>1. \end{array} \end{array} \end{array}$$

The configuration matrices *W*_*l*_, *l* = 0, 1, 2 are given by


$$\begin{array}{l} W_{0}=\left[\begin{array}{c} \begin{array}{cccccccc} -5 & 1 & 1 & 0 & 1 & 1 & 1 & 0\\ 1 & -5 & 0 & 1 & 1 & 1 & 0 & 1\\ 1 & 0 & -4 & 1 & 0 & 0 & 1 & 1\\ 0 & 1 & 1 & -4 & 1 & 0 & 1 & 0\\ 1 & 1 & 0 & 1 & -6 & 1 & 1 & 1\\ 1 & 1 & 0 & 0 & 1 & -3 & 0 & 0\\ 1 & 0 & 1 & 1 & 1 & 0 & -5 & 1\\ 0 & 1 & 0 & 0 & 1 & 0 & 1 & -4 \end{array} \end{array}\right],\\ W_{1}=\left[\begin{array}{c} \begin{array}{cccccccc} -4 & 0 & 0 & 1 & 1 & 0 & 1 & 1\\ 0 & -2 & 1 & 1 & 0 & 0 & 0 & 0\\ 0 & 1 & -4 & 1 & 1 & 1 & 0 & 0\\ 1 & 1 & 1 & -3 & 0 & 0 & 0 & 0\\ 1 & 0 & 1 & 0 & -4 & 1 & 0 & 1\\ 0 & 0 & 1 & 0 & 1 & -2 & 0 & 0\\ 1 & 0 & 0 & 0 & 0 & 0 & -1 & 0\\ 1 & 0 & 0 & 0 & 1 & 0 & 0 & -2 \end{array} \end{array}\right],\\ W_{2}=\left[\begin{array}{c} \begin{array}{cccccccc} -1 & 1 & 0 & 0 & 0 & 0 & 0 & 0\\ 1 & -2 & 0 & 1 & 0 & 0 & 0 & 0\\ 0 & 0 & 0 & 0 & 0 & 0 & 0 & 0\\ 0 & 1 & 0 & -3 & 1 & 0 & 0 & 1\\ 0 & 0 & 0 & 1 & -3 & 1 & 0 & 1\\ 0 & 0 & 0 & 0 & 1 & -1 & 0 & 0\\ 0 & 0 & 0 & 0 & 0 & 0 & 0 & 0\\ 0 & 0 & 0 & 1 & 1 & 0 & 0 & -2 \end{array} \end{array}\right]. \end{array}$$

The controller is designed as


(15) $$\begin{array}{l} u_{ik}=-\alpha_{i}e_{ik}(t)-sign(e_{ik}(t))(\beta_{i}+\sum_{l=0}^{2}|e_{ik}(t-\tau_{l})|\\ \;+\frac{D_{v}}{T_{v}}\delta_{i}|e_{ik}(t){|}^{\eta }), \end{array}$$

where η = 2 > 1, the remainder parameters are given by


$$\begin{array}{l} \alpha =\left[\begin{array}{ccc} 2 & 3 & 4\\ 2 & 3 & 4\\ 2 & 3 & 4\\ 2 & 3 & 4\\ 2 & 3 & 4\\ 2 & 3 & 4\\ 2 & 3 & 4\\ 2 & 3 & 4 \end{array}\right],\beta =\left[\begin{array}{ccc} 5 & 5 & 5\\ 5 & 5 & 5\\ 5 & 5 & 5\\ 5 & 5 & 5\\ 5 & 5 & 5\\ 5 & 5 & 5\\ 5 & 5 & 5\\ 5 & 5 & 5 \end{array}\right],r=\left[\begin{array}{ccc} 1 & 1 & 1\\ 1 & 1 & 1\\ 1 & 1 & 1\\ 1 & 1 & 1\\ 1 & 1 & 1\\ 1 & 1 & 1\\ 1 & 1 & 1\\ 1 & 1 & 1 \end{array}\right],\\ \delta =\left[\begin{array}{ccc} 12 & 12 & 12\\ 12 & 12 & 12\\ 12 & 12 & 12\\ 12 & 12 & 12\\ 12 & 12 & 12\\ 12 & 12 & 12\\ 12 & 12 & 12\\ 12 & 12 & 12 \end{array}\right]. \end{array}$$

**Figure 1:**
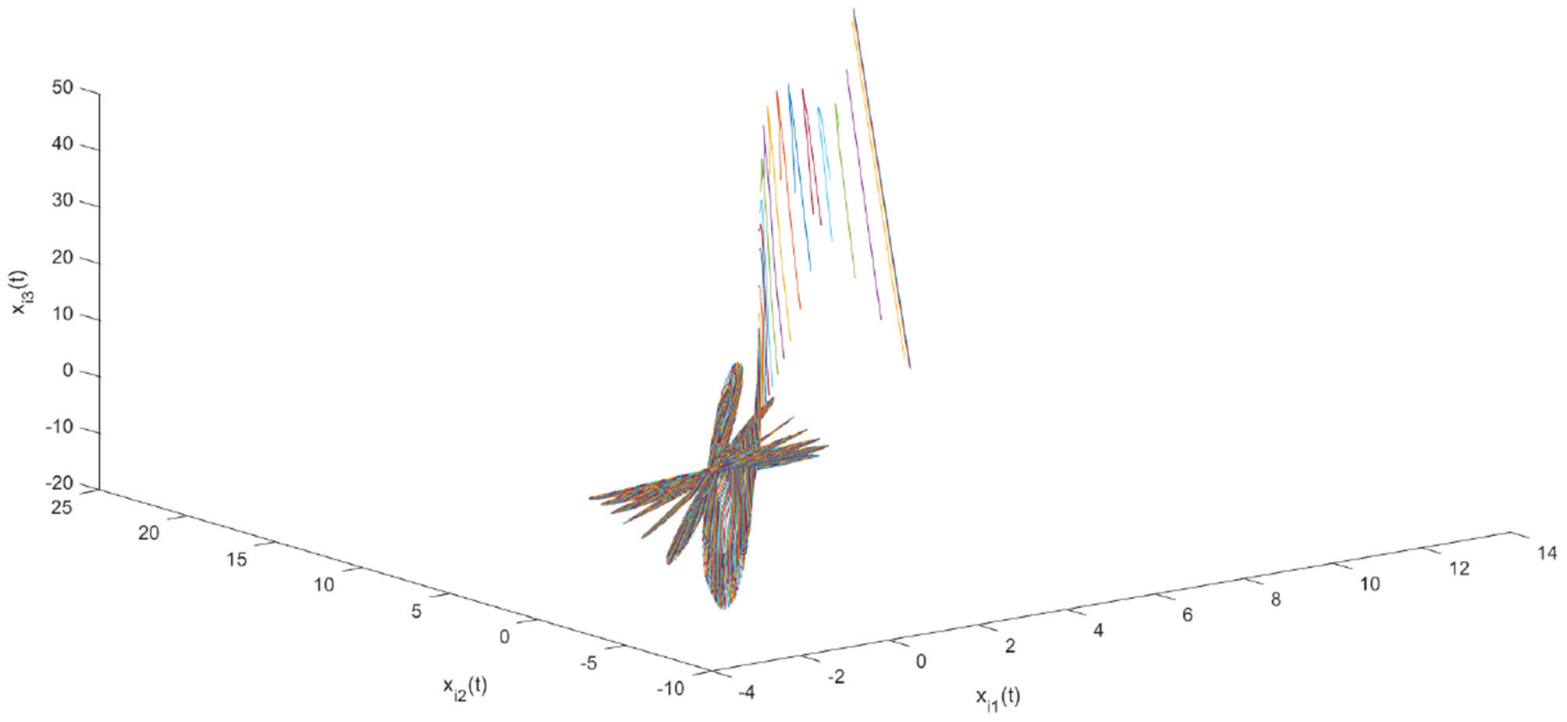
The phase curves of the drive system in three-dimensional neurons.

**Figure 2:**
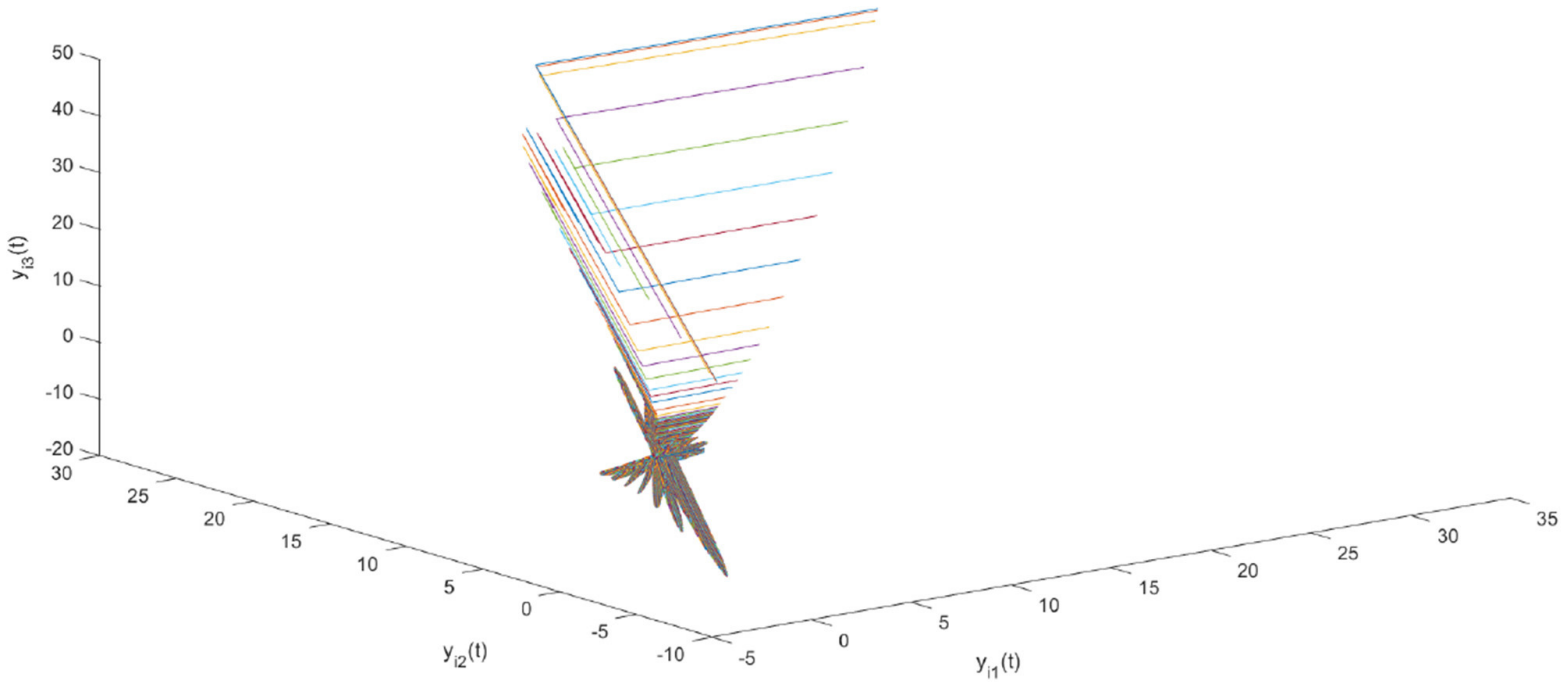
The phase curves of the response system without the controller in three-dimensional neurons.

**Figure 3:**
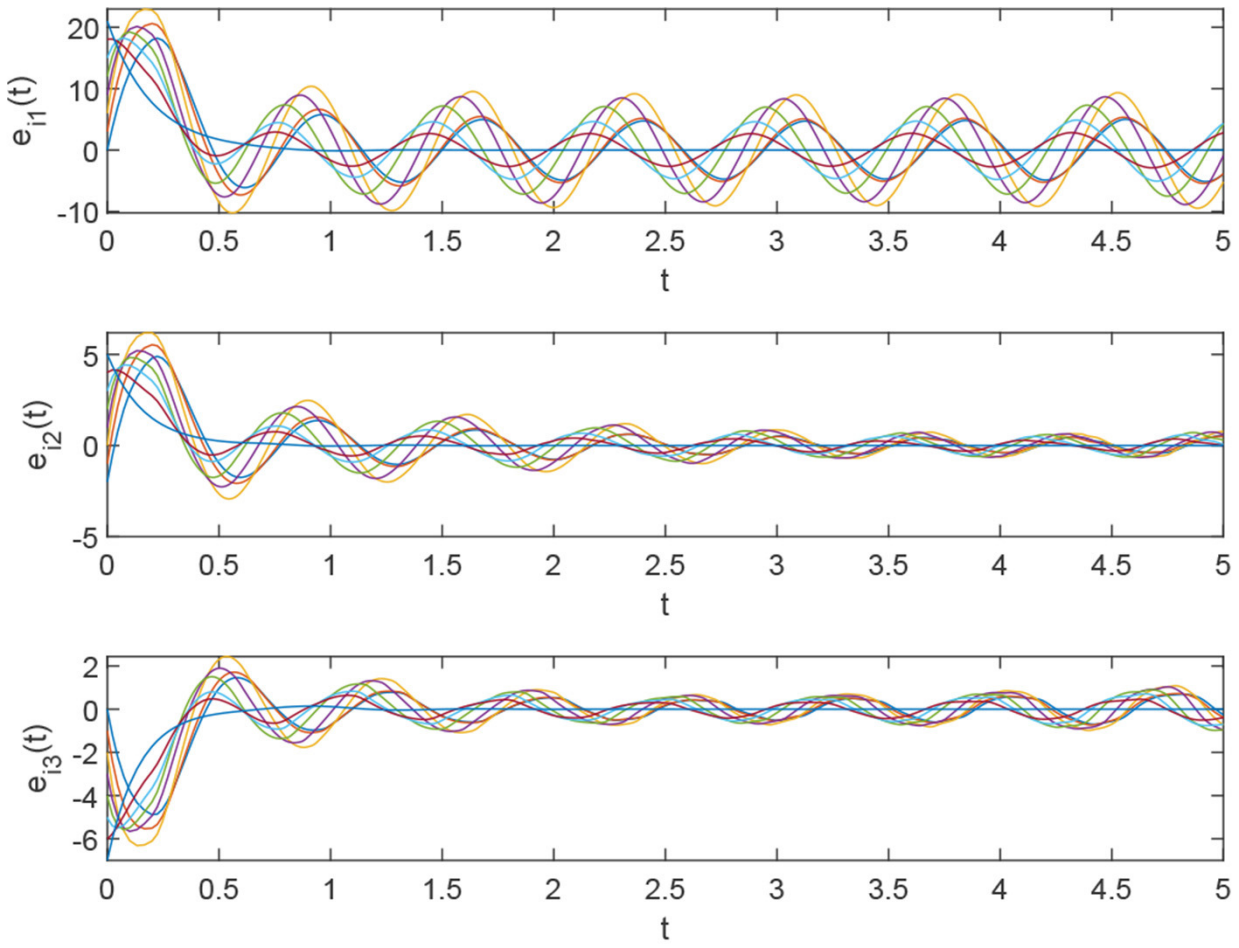
The error system of drive-response system (15) without the controller.

**Figure 4:**
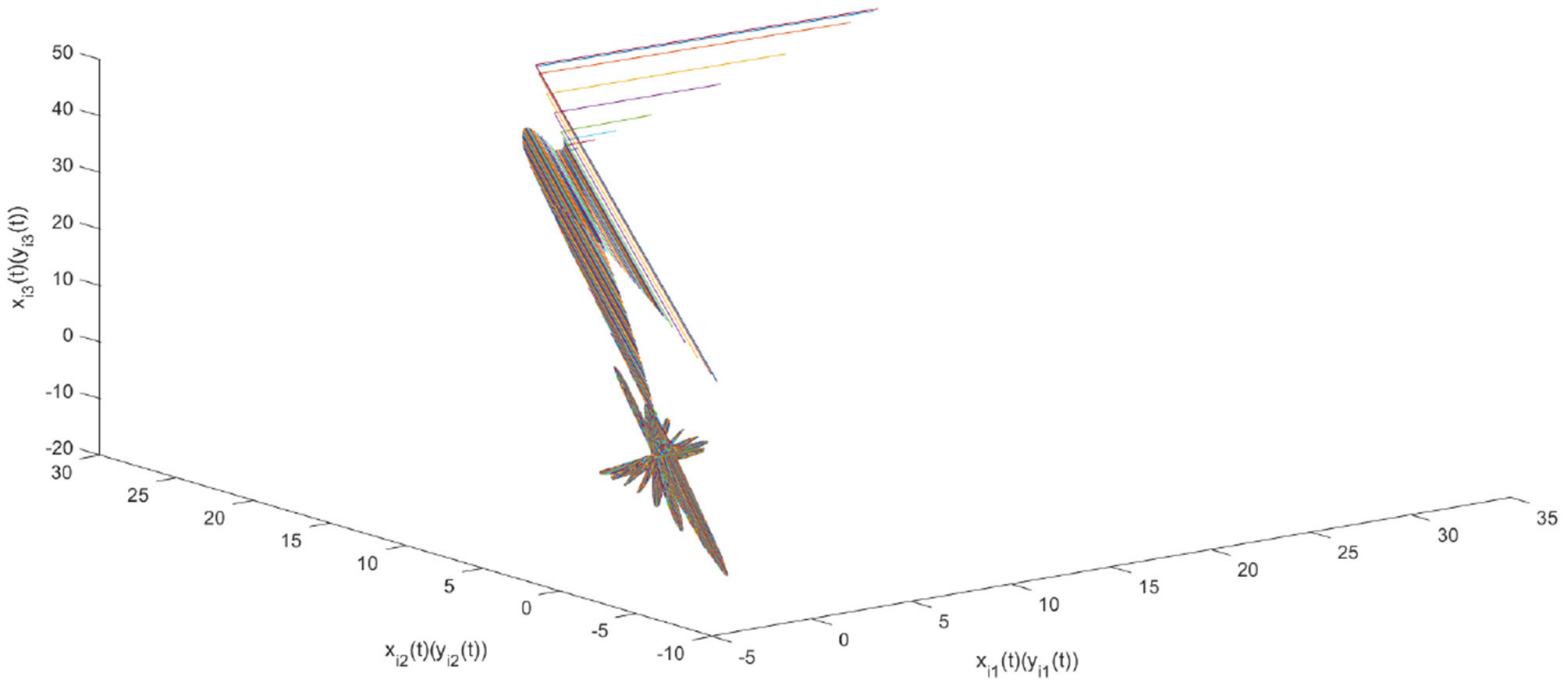
The phase curves of drive-response system (15) with the controller.

**Figure 5:**
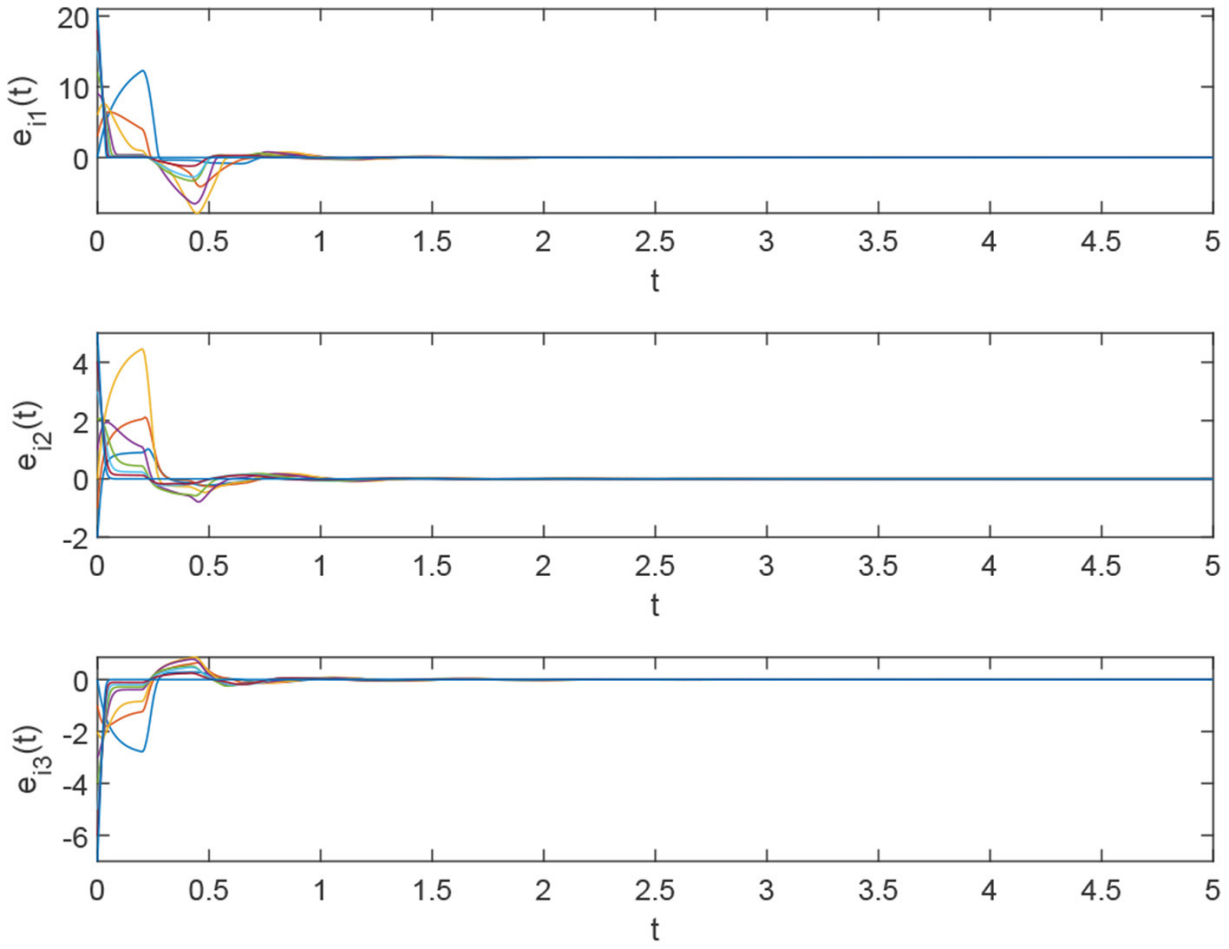
The error system of drive-response system (15) with the controller.

Using the parameters of controller (15), we have λ = 12, *a* = 4, *b* = 4, *and D*_*v*_ = 2, the synchronization is realized within the predefined time *T*_*v*_ = 2. Figures 1, 2 describe the phase curves of the drive-response system in three-dimensional neurons without the controller. Figure 3 shows the error state trajectory of drive-response system (14) without the controller. Figure 4 describes the phase curves of the drive-response system in three-dimensional neurons with the controller. Figure 5 shows the error state trajectory of drive-response system (14) with controller (15).

**Figure 6:**
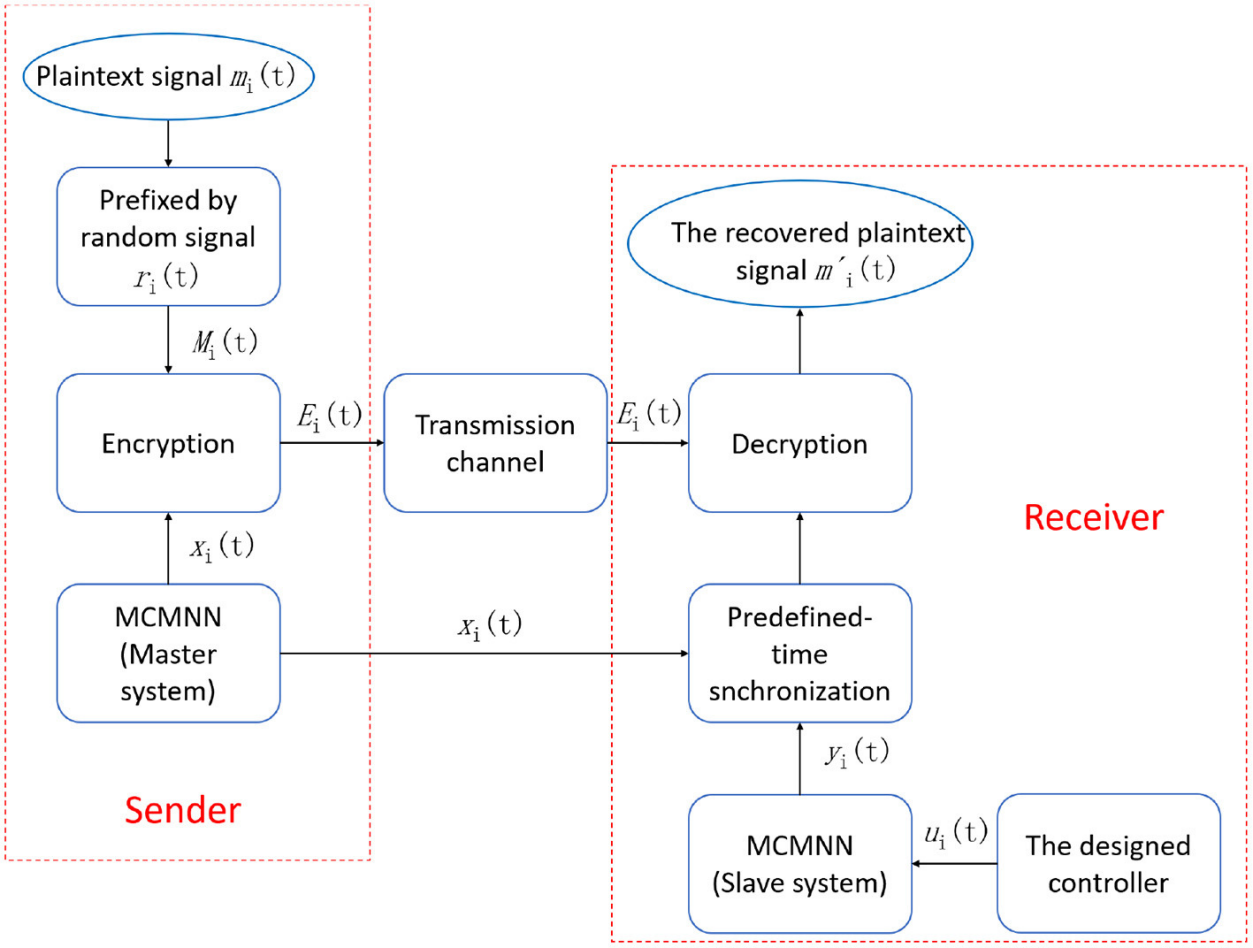
The schematic diagram of the proposed secure communication scheme.

**Example 2:** In this example, we give the secure communication scheme based on the predefined-time synchronization of drive-response systems. Figure 6 shows the schematic diagram of the proposed secure communication scheme. It is worth noting that the transmitted signals are superimposed on a single point three-dimensional neuron of the MCMNN. We consider the following MCMNN of single point form as the drive system:


(16) $$\begin{array}{l} \begin{array}{c} {ẋ}_{1i}\left(t\right)=-c_{i}x_{1i}\left(t\right)+\overset{n}{\underset{q=1}{\sum }}a_{iq}\left(x_{1i}\left(t\right)\right){ḡ}_{q}\left(x_{1i}\left(t\right)\right)\\ \;+\overset{n}{\underset{q=1}{\sum }}b_{iq}\left(x_{1i}\left(t-\tau_{0}\right)\right)g_{q}\left(x_{1i}\left(t-\tau_{0}\right)\right)\\ \;+\sigma \overset{N}{\underset{j=1}{\sum }}w_{1j}^{0}\Gamma x_{ji}\left(t\right)+\sigma \overset{m}{\underset{l=1}{\sum }}\overset{N}{\underset{j=1}{\sum }}w_{1j}^{l}\Gamma x_{ji}\left(t-\tau_{l}\right), \end{array} \end{array}$$

and


$$M_{i}(t)=\{\begin{array}{c} r_{i}(t),0\leq t<T_{v},\\ m_{i}(t-T_{v}),t\geq T_{v}. \end{array}$$

where


$$\{\begin{array}{c} r_{1}(t)=r\;and\;(-1,1),\\ r_{2}(t)=r\;and\;(-3,3),\\ r_{3}(t)=r\;and\;(-2,2), \end{array}\;\left\{ \begin{array}{l} m_{1}(t)=0.5\sin(2t)+0.3\cos(0.5t),\\ m_{2}(t)=-\sin(3t)+2\cos(1.2t),\\ m_{3}(t)=\sin(3t)-2\cos(3t). \end{array}\right.$$

**Figure 7:**
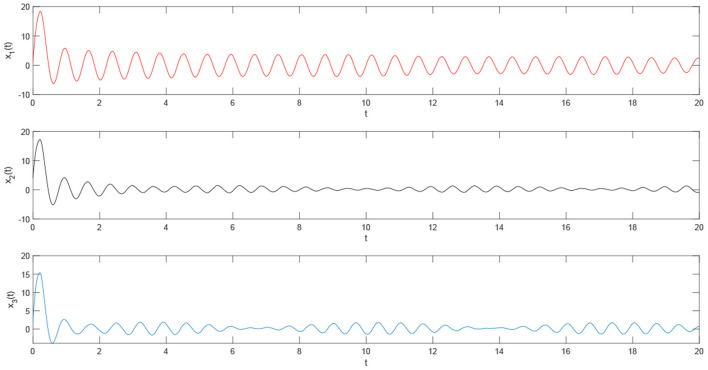
The state curve of single three-dimensional system (16).

**Figure 8:**
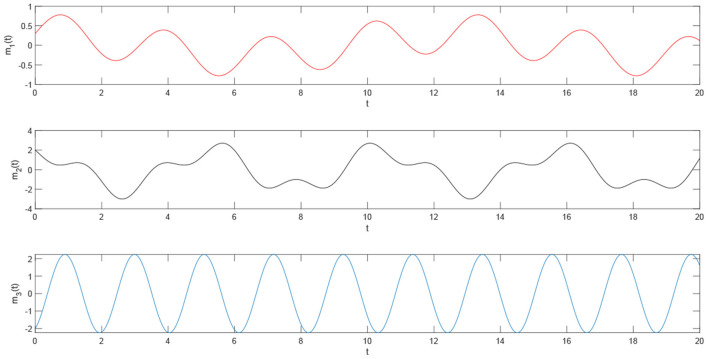
The time trajectory curve of the plaintext signal.

**Figure 9:**
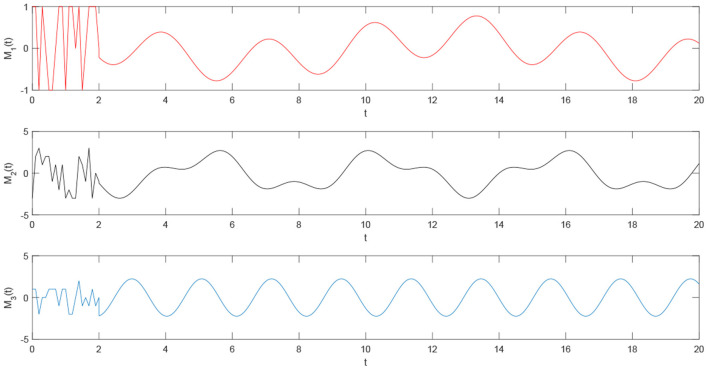
The time trajectory curve of mixed signal by the plaintext signal and random signal.

**Figure 10:**
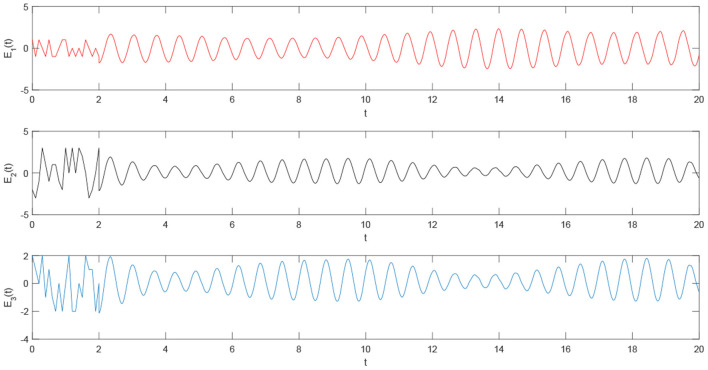
The time trajectory curve of the encrypted signal.

The remaining parameters are given as they are in Example 1. Figures 7–10 illustrate the state trajectories of *x*_1*i*_(*t*), *m*_*i*_(*t*), *M*_*i*_(*t*), and *E*_*i*_(*t*), *i* = 1, 2, 3. The initial values of drive system (17), *m*_*i*_(*t*), *r*_*i*_(*t*), and *M*_*i*_(*t*) can only be known by the sender. The common keys of the sender and receiver are *c*_*i*_ and *T*_*i*_ and the predefined synchronization time *T*_*v*_. After the receiver receives the secret keys and the encrypted signal *E*_*i*_(*t*), *i* = 1, 2, 3, the receiver generates the response system as follows:


(17) $$\begin{array}{l} \begin{array}{c} {ẏ}_{1i}\left(t\right)=-c_{i}y_{1i}\left(t\right)+\overset{n}{\underset{q=1}{\sum }}a_{iq}\left(y_{1i}\left(t\right)\right){ḡ}_{q}\left(y_{1i}\left(t\right)\right)\\ \;+\overset{n}{\underset{q=1}{\sum }}b_{iq}\left(y_{1i}\left(t-\tau_{0}\right)\right)g_{q}\left(y_{1i}\left(t-\tau_{0}\right)\right)\\ \;+\sigma \overset{N}{\underset{j=1}{\sum }}w_{1j}^{0}\Gamma y_{ji}\left(t\right)+\sigma \overset{m}{\underset{l=1}{\sum }}\overset{N}{\underset{j=1}{\sum }}w_{1j}^{l}\Gamma y_{ji}\left(t-\tau_{l}\right)+u_{i}\left(t\right), \end{array} \end{array}$$

where the parameters are given as in Example 1 too. Since *T*_*v*_ = 2, we have *x*_1*i*_(*t*) = *y*_1*i*_(*t*), *i* = 1, 2, 3, *t* ≥ 2. The receiver can decrypt the encrypted signal by calculating the following formula:


$$\begin{array}{l} \begin{array}{c} m_{i}^{\prime }\left(t\right)=E_{i}\left(t+2\right)-y_{1i}\left(t+2\right),\\ \;=M_{i}\left(t+2\right)+x_{1i}\left(t+2\right)-y_{1i}\left(t+2\right),\\ \;=M_{i}\left(t+2\right)\\ \;=m_{i}\left(t\right),t\geq 0. \end{array} \end{array}$$

**Figure 11:**
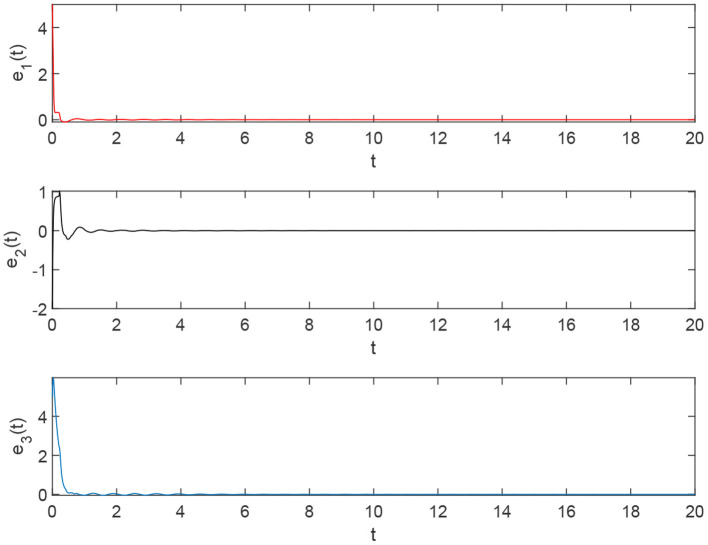
The error system of drive-response systems (17) and (18) with the controller.

Figure 11 illustrates the state trajectories of the error system under the controller.

## 6. Conclusion and Prospects

We investigated the predefined-time synchronization of coupled memristive neural networks with multi-links coupled forms; the multi-links performance increased the complexity and the instability of systems. The predefined-time stability theorem and the effective controller are given to guarantee predefined-time synchronization of drive-response systems based on differential inclusion theory and the concept of set-valued mapping. Further, we designed an effective secure communication scheme based on predefined-time synchronization of drive-response systems. Undeniably, compared with some mature secure communication schemes, the secure communication schemes in this section are also relatively shallow. However, the related research results are expected to provide a new perspective for the research of secure communication. Finally, numerical simulation of predefined-time synchronization and an example of secure communication are shown to verify the effectiveness of theoretical research. In the future, a more expansibility secure communication scheme based on predefined-time stability will be designed to optimize encryption and selective encryption schemes needed by people.

## Data Availability Statement

The original contributions presented in the study are included in the article/supplementary material, further inquiries can be directed to the corresponding author.

## Author Contributions

HZ: formal analysis, funding acquisition, validation, and writing-original draft. LL, SN, and MZ: funding acquisition and supervision. CC: methodology and supervision: QW and AL: data curation. All authors contributed to the article and approved the submitted version.

## Funding

The work is supported by the National Natural Science Foundation of China (Grant Nos. 62103165, 12172201, and 62032002), the Natural Science Foundation of Shandong Province (Grant Nos. ZR2021MF072, ZR2020MA054, and ZR2021MF090), and the 111 Project (Grant No. B21049).

## Conflict of Interest

The authors declare that the research was conducted in the absence of any commercial or financial relationships that could be construed as a potential conflict of interest.

## Publisher's Note

All claims expressed in this article are solely those of the authors and do not necessarily represent those of their affiliated organizations, or those of the publisher, the editors and the reviewers. Any product that may be evaluated in this article, or claim that may be made by its manufacturer, is not guaranteed or endorsed by the publisher.
